# The prognostic value of peripheral CD4^+^CD25^+^ T lymphocytes among early stage and triple negative breast cancer patients receiving dendritic cells-cytokine induced killer cells infusion

**DOI:** 10.18632/oncotarget.5534

**Published:** 2015-10-05

**Authors:** Qing-Kun Song, Jun Ren, Xin-Na Zhou, Xiao-Li Wang, Guo-Hong Song, Li-Jun Di, Jing Yu, Amy Hobeika, Michael A. Morse, Yan-Hua Yuan, Hua-Bing Yang, Herbert Kim Lyerly

**Affiliations:** ^1^ Beijing Key Laboratory of Therapeutic Cancer Vaccines, Capital Medical University Cancer Center, Beijing Shijitan Hospital, Beijing 10038, China; ^2^ Department of Medical Oncology, Peking University Cancer Hospital & Institute, Beijing 100142, China; ^3^ Department of Surgery, Duke University Medical Center, Durham, NC 27710, United States; ^4^ Department of Medicine, Duke University Medical Center, Durham, NC 27710, United States

**Keywords:** CD4^+^CD25^+^ T lymphocyte, early stage breast cancer, triple negative breast cancer, prognosis, adoptive cell therapy

## Abstract

**Objective:**

This study aimed to assess the prognostic value of CD4^+^CD25^+^ T lymphocyte in peripheral blood among breast cancer patients treated with adoptive T lymphocytes immunotherapy.

**Methods:**

217 patients participated in the follow-up study. CD4^+^CD25^+^ proportion was measured by flow cytometry in peripheral T cells. The median survival was estimated by Kaplan-Meier curve, Log-rank test and Cox hazard proportion regression model, between groups of CD4^+^CD25^+^ proportion more than 5% and less than or equal to 5% in peripheral T cells.

**Results:**

Peripheral CD4^+^CD25^+^ T lymphocytes had not a relationship with progression-free survival. It was featured that above 5% peripheral CD4^+^CD25^+^ proportion of T cells was related with the median overall survival by a shorten of 51 months (*p* < 0.05) with the HR 1.65 (95%CI 1.04, 2.62). Above 5% CD4^+^CD25^+^proportion of T cells produced the HR to be 1.76 (95%CI 1.07, 2.87) In stage 0-II patients, and 3.59 (95%CI 1.05, 12.29) in triple negative breast cancer patients.

**Conclusion:**

Cellular immunity restoration recovered by adoptive T cell infusions which resulted in less proportion of peripheral CD4^+^CD25^+^T lymphocytes could be a potential prognostic indicator among early stage and triple negative patients.

## INTRODUCTION

Breast cancer (BC) is the second most common cancer worldwide, with an estimated 1.67 million new cases diagnosed in 2012 (25% of all cancers) [1]. By far, BC is the most common female cancer in China, with 187213 new cases in 2012. However, in 2012 the 5-year prevalent cases were 697327 [1]. The gap between incident number and prevalent number indicated a big health care demand for Chinese BC patients that the more prevalent patients the more health services required. Currently, the screening program was inadequate to reduce BC burden in China [2].

Current clinical evidences showed that cancer immunotherapy has been becoming the promising approaches which enhance the microenvironment anti-cancer ability and reject the T cell exhausting [3]. The mechanism of immunotherapies included stimulation and redirection of the cellular immune responses to cancer cells and the action of cell lysis [4]. Dendritic cells (DC) are the antigen-presenting cells and able to promote the generation of helper T cells and cytotoxic T cells. DC acts as effective T-cell stimulators and induces a tumor-specific immune response. Cytokine induced killer (CIK) cells are *ex vivo*-expanded T lymphocytes—a subset of T lymphocytes with a natural killer T-cell phenotype expressing both the CD56 and the CD3 markers, present non-histocompatibility complex cytotoxicity against target cells [5, 6]. Our previous study indicated that high-dose chemotherapy combined with dendritic cells-cytokine induced killer cells (DC-CIK) improved both progression-free and overall survival in metastatic BC patients [7]. Continuous DC-CIK infusions were capable of improving clinical efficacy, physical activity and cellular immunity among advanced malignancy patients [8]. Recently such combination of chemotherapy and adoptive T cells generated the encouragingclinical benefits for young triple negative breast cancer (TNBC) patients [9]. In a meta-analysis, DC-CIK cell therapy markedly prolonged survival time by 1-year more, enhanced immune function by NK cell increase, and improved the clinical efficacy [10]. Increasing evidences suggest that DC-CIKs can increase the survival and quality of life among BC patients. It was emerging to explore the significant predictive and prognostic biomarkers for immunotherapy to prospectively evaluate clinical treatments.

T lymphocyte subtypes were observed to be associated with BC patients' survival [7]. Increased level of tumor-infiltrating regulatory CD4^+^CD25^+^ T lymphocytes was related with lymph node metastasis among BC patients [11]. Intratumoral CD4^+^CD25^+^ regulatory T cell number was a prognostic factor for progression-free and overall survival for BC patients with neoadjuvant and adjuvant chemotherapy [12, 13]. However, it is not feasible to obtain re-biopsies for T cell infiltration tests among advanced BC patients. Thus there was an increasing demand that whether alternatively monitoring the circulating T lymphocytes to predict the prognosis of cancer patients rather than tissue biopsy tests. We have previously reported that peripheral blood CD8^+^CD28^−^ subsets could be used as the independently prognostic factor in metastatic BC [14]. There are more and more BC patients receiving DC-CIK infusion treatment worldwide but the markers of predicting the clinical responses and outcomes were few determined. Additionally, triple negative BC patients (TNBC) were reported to have special immunological characteristics by elevated expression of immune genes, suggesting the potential bebefuts from immunotherapy [15]. This study aimed to investigate the prognostic effects of peripheral blood but not tissue infiltrating CD4^+^CD25^+^ T cells for BC patients receiving DC-CIK infusions, to testify the feasibility and values of T cell subsets proportion as substitute biomarkers for breast cancer patients.

## RESULTS

BC patients with more than 5% CD4^+^CD25^+^ proportion in peripheral T cells were younger than patients with the proportion less than or equal to 5%, but the difference was not significant (Table 1). Between the two groups of peripheral CD4^+^CD25^+^proportion of T cells, patients had not significant differences in pathological stage, estrogen receptor, HER2+ and molecular subtypes (Table 1). Patients with a high proportion had the positive progesterone receptor being 14.4% higher (*p* < 0.05, Table 1). Clinical treatments including chemotherapy, radiotherapy and endocrine-therapy were not different between low and high CD4^+^CD25^+^ proportion of peripheral T cells (Table 1).

**Table 1:** Characteristics for BC patients with low and high CD4^+^CD25^+^ T lymphocytes

	Proportion of cells		*p*
	less than or equal to 5%	more than 5%	
Age, median (interquartile range)*	51.43 (41.56, 56.93)	46.34 (41.18, 56.07)	0.07
Stage, n (%)			
≤I	16 (16.7)	8 (7.4)	0.11
II	41 (42.7)	47 (43.5)	
≥III	39 (40.6)	53 (49.1)	
Estrogen receptor, n (%)			0.82
Negative	41 (43.6)	47 (45.2)	
Positive	53 (56.4)	57 (54.8)	
Progesterone receptor, n (%)			0.04
Negative	39 (41.9)	58 (56.3)	
Positive	54 (58.1)	45 (43.7)	
HER2+			0.52
–	34 (35.8)	45 (43.7)	
+	17 (17.9)	21 (20.4)	
++	24 (25.3)	21 (20.4)	
+++	20 (21.1)	16 (15.5)	
Molecular subtype, n (%)			0.50
Luminal A	37 (50.7)	42 (50.0)	
Luminal B	8 (11.0)	9 (10.7)	
HER2+	12 (16.4)	8 (9.5)	
Triple Negative	16 (21.9)	25 (29.8)	
Chemotherapy, n (%)			0.05
Primary line	48 (59.3)	43 (55.2)	
Second line	13 (16.0)	24 (30.8)	
≥Third line	20 (24.7)	11 (14.1)	
Radiotherapy, n (%)			0.42
No	61 (59.2)	66 (62.3)	
Yes	42 (40.8)	40 (37.7)	
Endocrine-therapy, n (%)			
No	53 (50.5)	63 (56.3)	0.39
Yes	52 (49.5)	59 (43.7)	

* Wilcoxon-Rank Sum test

**Figure 1:**
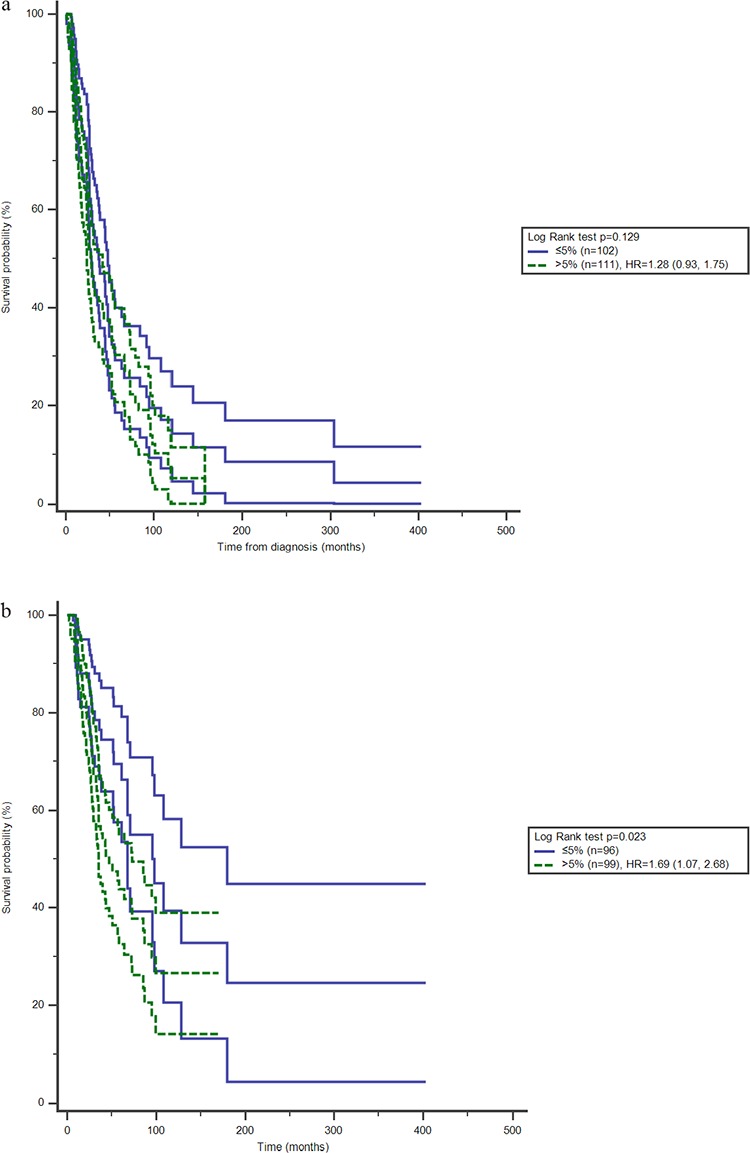
Effects of CD4^+^ CD25^+^ proportion of T cells on survival with 95%CI among BC patients, a. progression-free survival, b. overall survival

Peripheral CD4^+^CD25^+^ proportion of T cells was not related with BC patients' progression-free survival, the median survival time being 37.0 months in patients with the proportion less than or equal to 5% and 28.0 months in patients with the proportion more than 5% (*p* > 0.05, Figure 1a). CD4^+^CD25^+^ proportion in peripheral blood had a significant association with overall survival (Figure 1b). Median survival length was 97.8 months among patients with proportion less than or equal to 5%, in contrast with 46.8 months among patients with CD4^+^CD25^+^proportion more than 5% (*p* = 0.02, Table 2). More than 5% CD4^+^CD25^+^proportion in peripheral blood increased the relative risk of death to 1.65 (95%CI 1.04, 2.62) with confounder adjustment (Table 2). Positive progesterone receptor and endocrine-therapy increased the overall survival too (Table 2).

**Table 2:** Univariate and multivariate analysis between interested factors and overall survival

	Median survival (months)	*p* value of Log-Rank test	HR_crude_ (95%CI)	HR_adjusted_ (95%CI)*
CD4^+^CD25^+^ proportion of T cells				
≤5%	97.8	0.023	1.00	1.00
>5%	46.8		1.69 (1.07, 2.68)	1.65 (1.04, 2.62)
PR				
Negative	35.2	<0.001	1.00	1.00
Positive	96.1		0.41 (0.26, 0.63)	0.55 (0.32, 0.96)
Chemotherapy				
Primary line	87.2	0.104	1.00	1.00
Second line	43.8		1.65 (0.98, 2.79)	1.73 (0.96, 3.58)
≥Third line	38.7		1.56 (0.89, 2.74)	1.62 (0.91, 3.46)
Radiotherapy				
No	73	0.205	1.00	1.00
Yes	58.4		1.31 (0.86, 2.01)	1.68 (0.90, 2.87)
Endocrine-therapy				
No	52.4	0.028	1.00	1.00
Yes	94.7		0.63 (0.42, 0.96)	0.49 (0.26, 0.91)

* further adjusted age

**Figure 2:**
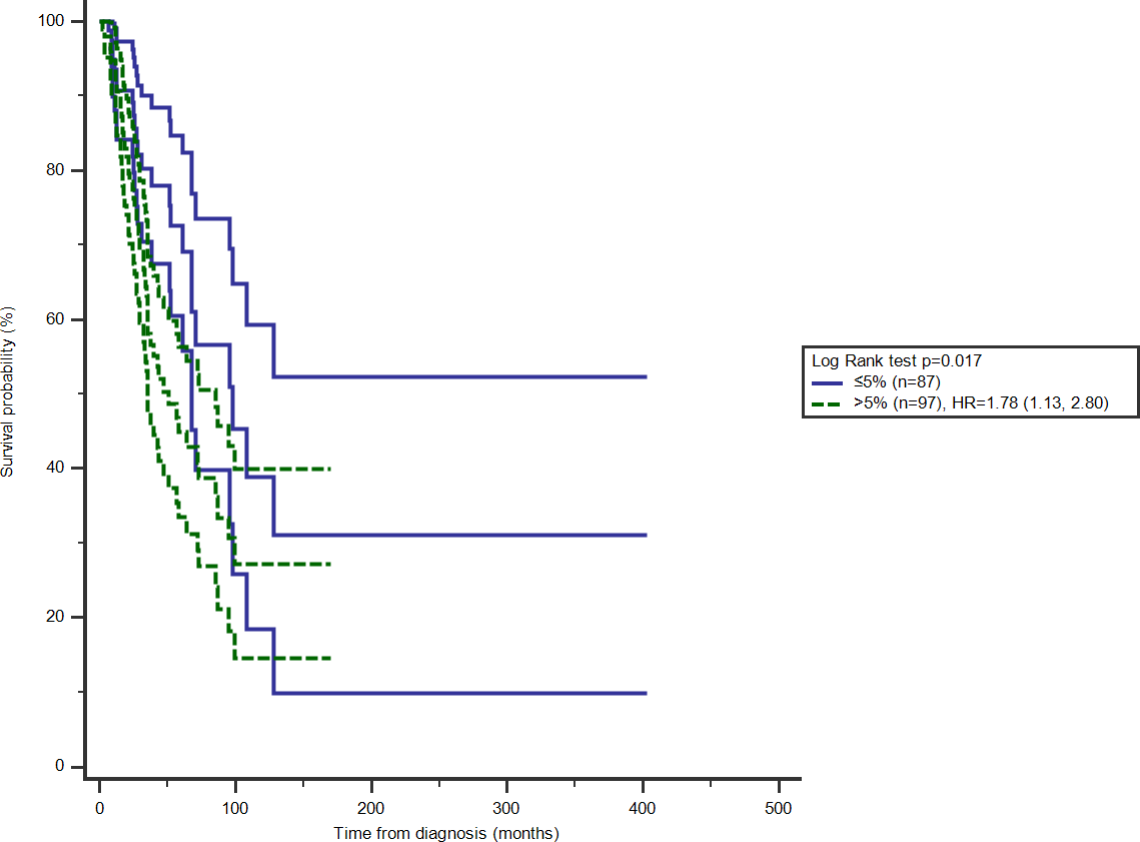
Effect of CD4^+^CD25^+^proportion of T cells on overall survival with 95%CI among BC patients in stage ≤ II

**Figure 3:**
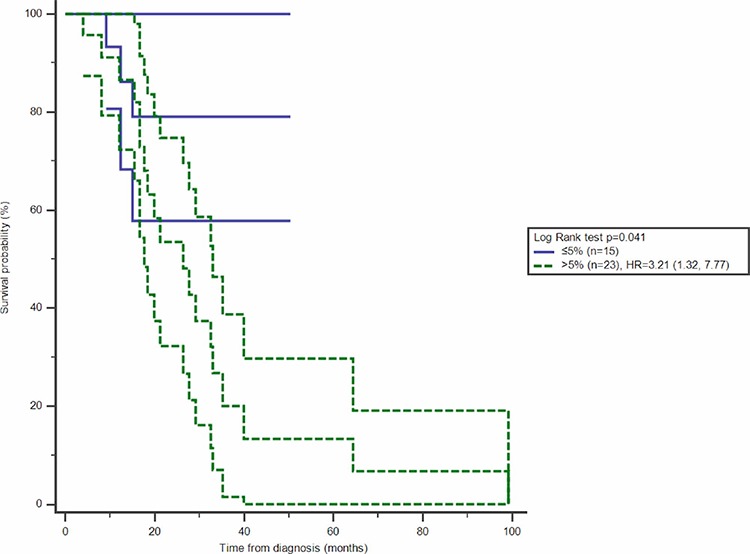
Effect of CD4^+^CD25^+^proportion of T cells on overall survival with 95%CI among TNBC patients

With stratification analysis, the significantly survival difference was observed among the patients in early stage, HR of more than 5% CD4^+^CD25^+^ proportion of T cells increasing to 1.76 (95%CI 1.07, 2.87) in stage ≤ *II* (Figure 2, Table 3). Stratified with molecular subtypes, CD4^+^CD25^+^ T cells were related with overall survival among TNBC patients, more than 5% proportion of peripheral T cells increasing the HR to be 3.59 (95%CI 1.05, 12.29) (Figure 3, Table 3).

**Table 3:** Subgroup analysis between CD4^+^CD25^+^ T cell and overall survival

	cell proportion	Median survival (months)	*p*	HR*	95%CI
Stage					
≤II	≤5%	97.8	0.02	1.00	
	>5%	50.9		1.76	1.07, 2.87
>II	≤5%	67.7	0.09		
	>5%	34.3		1.91	0.93, 3.93
Molecular subtype					
Luminal A	≤5%	108.0	0.46		
	>5%	NA		0.73	0.30, 1.79
Luminal B	≤5%	70.8	0.10		
	>5%	33.9		5.90	0.64, 54.47
HER2+	≤5%	38.7	0.73		
	>5%	16.1		1.23	0.31, 4.85
TNBC	≤5%	NA	0.04		
	>5%	26.3		3.59	1.05, 12.29

* adjusting age, PR, chemotherapy, endocrine-therapy and radiotherapy.

## DISCUSSION

The prognostic significance of tissue tumor-associated CD4^+^CD25^+^ T cells was observed in many previous studies. Liu et al. reported high tumor-infiltration of Forkhead/winged helix transcription factor 3 (FOXP3)^+^ CD4^+^CD25^+^ regulatory T cell was related with shorter survival among BC patients and high amount cells produced a 1.90-fold high risk of death and a 1.53-fold high risk of progression [16]. Another study indicated high level of intratumoral CD4^+^CD25^+^ regulatory T cells was even related with a 5.93-fold high risk of death [13]. Increased intratumoral CD4^+^CD25^+^ regulatory T cells was related with shorter survival of BC patients, especially basal-like subtype and the patients with hormone therapy [17]. Even among early BC patients, high tumor-infiltrating CD4^+^CD25^+^ regulatory T cells were associated with earlier recurrence, lymph node metastasis, and expression of p53 and K67 [11]. Tumor infiltration status of CD4^+^CD25^+^ regulatory T cell was prognostic indicator of disease-free and overall survival. Proportion of peripheral blood CD4^+^CD25^+^ regulatory T cells were also related with BC pathological characteristics that advanced stages were more likely to present higher level cells [18]. The percentage of CD4^+^CD25^+^ regulatory T in the peripheral blood was significantly higher in BC patients and significantly ascended by the stage [19]. For the difficulties of accessing to tissue biopsies during treatment process, detection of peripheral CD4^+^CD25^+^ T cells was an alternative to predict the prognosis.

Many molecules involved in the mechanism pathway of CD4^+^CD25^+^ regulatory T cells. Bulk transfer of lymphocytes containing suppressor lymphocyte subsets, such as regulatory T cells, was reported to damage anti-tumor effect [20]. Infusion of autologous infiltrating lymphocytes depleting CD4^+^CD25^+^ regulatory T cells to BC murine, was capable of increasing activation and proliferation of CD4^+^ and CD8^+^ T cells, enhancing IL-4 and IFN-γ secretion, postponing tumor growth and prolonging survival [20]. Basal-like subtype of BC had a higher CXCR4 expression in CD4^+^CD25^+^ regulatory T cells and CXCR4 expression played an important role of promoting CD4^+^CD25^+^ regulatory T recruitment and suppressing immune response [17]. Immunopositivity of p53 and K67 were correlated with high CD4^+^CD25^+^ regulatory T infiltration [11]. Generation of CD4^+^CD25^+^ regulatory T cells is known to play a major role in progression and modulation of the immune-escape mechanisms in cancers. These cells express FOXP3 and Cytotoxic T-lymphocyte antigen-4 (CTLA-4), as a negative regulatory molecule: BC patients had increased levels FOXP3 and CTLA-4 [21]. CA15-3 was another molecule correlated with the percentage of CD4^+^CD25^+^ regulatory T cells in the peripheral blood [19]. Immune dysregulation was related with elevated level of functionally active CD4^+^CD25^+^ regulatory T cells in melanoma patients and patients with increased CD4^+^CD25^+^ regulatory T cells have higher risk of progression [22]. High percentage of CD4^+^CD25^+^ T cells had inhibitory effect on immune system and impaired the therapeutical actions.

FOXP3 is a marker for immunosuppressive CD4^+^CD25^+^ regulatory T cells. In TNBC patients, more than 15 FOXP3-positive CD4^+^CD25^+^ regulatory T per 10 high power fields in the peritumoral area was an independent prognostic factor for overall survival and progression free survival with hazard ratios of 2.4 (95% CI 1.0–5.6; *p* = 0.049) and 2.0 (95% CI 1.1–3.6; *p* = 0.032), respectively [23]. High FOXP3^+^ tumor infiltrated lymphocyte levels were strongly associated with prolonged recurrence-free survival among TNBC patients but not ER-negative cases [24]. Compared with Lunimal A, TNBC patients had higher levels of IL-5, IFN-γ, IFN-α2 and TNF-α, indicating the particular immune function [15]. Early stage of BC intended to have high level of IL-2 [25]. Higher average numbers of FoxP3^+^ cells were also significantly associated with larger tumor size [26]. The high immunity in early stage might explain the prognostic effects of CD4^+^CD25^+^ T lymphocytes in stage 0 I and II. The particular immune profiles in TNBC and early stage cases might induce the prognostic effect of CD4^+^CD25^+^ T cells and the potential immunotherapeutic targets among these patients.

Depletion of CD4^+^CD25^+^ T cells was a potential strategy for cancer treatment. Low dose cyclophosphamide had inhibitory effect to CD4^+^CD25^+^ T cells. Radiation combining low dose cyclophosphamide and anti-CD25 antibody, compared irradiation alone significantly increased the effector T cells, survival rate, and suppressed tumor growth [27]. In lung cancer rice model, cyclophosphamide could inhibit lung metastasis, reduce tumor weight and volume, and decrease the CD4^+^CD25^+^ T cells in spleen and metastasis foci [28]. Meanwhile, cyclophosphamide decreased the levels of IL-6, TGF-β, IL-23, IFN-γ, Foxp3, RORγt, JAK2 and STAT3 but increased SOCS3 level [28]. The depletion of CD4^+^CD25^+^ T cells might activate through SOCS/JAK-STAT pathway and inflammatory cytokine responses. Combination of therapeutic vaccine and CD4^+^CD25^+^ T depletion strategy prolonged survival and reduced progression in mice with pancreatic intraepithelial neoplasm, compared with vaccine alone [29]. To metastasized BC patients, metronomic cyclophosphamide treatment could reduce CD4^+^CD25^+^ T cells, induce stable tumor-specific T cell responses and improve disease stabilization and survival [30]. CD4^+^CD25^+^ T cell was a potential target for therapeutic strategy for malignant patients.

DC-CIK infusion was related with BC survival and improvement of immune function. *Ren* et al reported combined therapy of chemo and DC-CIK immunotherapy increased the progression-free and overall survival of metastatic BC [7]. DC co-cultured with CIK cells was capable of reducing the number and function of CD4^+^CD25^+^ T cells [31, 32]. One of our data also presented an improvement in immune function and BC clinical outcome from DC-CIK infusions [8].

Since there were increasingly evidences that cancers included immunogenetic or non-immunogenetic types, we were not able to discriminate the types for those BC patients. The T cell infiltration in tumor microenvironment was not identified as well. This will lead to further studies on the precise immune modulators and determine the choices of immunotherapy including therapeutic vaccines and cytokines combinations. The sample size in subgroups was limited for estimation and introduced false-negative findings possibly. Missing data in collection was another limitation in this study.

## PATIENTS AND METHODS

This study was approved by both Peking University Cancer Hospital and Beijing Shijitan Hospital Institutional Review Board. Written consent was obtained from all patients.

251 Patients were invited into the study between January 2007 and June 2011. All patients were eligible for receiving standard salvage chemotherapy and had at least an Eastern Cooperative Oncology Group performance status of 0–2 and a life expectancy at least three months. We excluded patients that had already received immunotherapies following chemotherapy. 34 cases failed to test CD4^+^CD25^+^ proportion of T cells in peripheral blood after DC-CIK therapy and were excluded. After recruitment, the included 217 subjects were followed up every three months though telephone interview and collected the data of progression and survival status till March 31, 2012. The start time of follow-up was defined as the diagnosis date and 7.8% cases were lost to followup finally. One cycle DC-CIKs treatment included three infusions and T lymphocyte detection was performed at the end of the first DC-CIK cycle.

Mononuclear cells were harvested from peripheral blood and expanded *in vitro*. For the induction of DC-CIKs, peripheral blood mononuclear cells were mobilized by G-CSF. Apheresis was performed using the COBE Spectra cell separator (COBE BCT, Lakewood, CO, USA) until CD34^+^ reaching ≥4.5 × 10^6^/kg. 25–50 ml of the apheresis product was co-cultured with IL-4, TNF-αand GM-CSF *in vitro* to generate autologous DCs. With adequate cell counts, the cultured cells were infused intravenously and cellular immunity in peripheral blood was measured by flow cytometry.

Two milliliters of heparinized peripheral venous blood was obtained from each patient. Whole blood (100 μl) was incubated in the dark with primary antibody at 4°C for 15 min. After hemolysis for 10 min, samples were centrifuged for 10 min at 1,500 rpm at room temperature, and then washed twice in PBS and subjected to flow cytometric analysis. Primary antibodies included: anti-CD3-PC5 (Beckman-Coulter), anti-CD4-FITC (Beckman-Coulter) and anti-CD25-PE (BeckmanCoulter).

Three-color flow cytometric analysis was performed to determine cell phenotypes. CD4^+^CD25^+^ T lymphocyte subset levels were reported as percentages of the total T lymphocytes in peripheral blood. Flow cytometry was performed using an FC500 (Beckman-Coulter), and CXP analysis software (Beckman-Coulter). Lymphocytes were gated by forward scatter versus side scatter. T lymphocytes were gated on CD3^+^ positive cells, and CD4^+^CD25^+^ T lymphocytes were gated on CD4^+^CD25^+^ cells in the lymphocyte gate. Analysis was set to collect 5,000 gated events.

### Statistical analysis

The median percentage of CD4^+^CD25^+^proportion of T cells was 5% in total peripheral T lymphocytes and set as cut-off point for two groups (≤5% vs. >5%). The statistical analyses were conducted under SPSS software 17.0 version. Age was analyzed by Wilcoxon-Rank Sum test between the two groups. Stage, HER2+ status, estrogen receptor, progesterone receptor, molecular subtypes, chemotherapy, radiotherapy and endocrine-therapy were analyzed through Chi-square test. Association between CD4^+^CD25^+^ proportion of T cells, progression-free and overall survival was estimated by Kaplan-Meier method with Log-rank test. The Kaplan-Meier survival curve and 95% confidence interval (95%CI) were displayed by MedCal software (https://www.medcalc.org/index.php). Cox hazard proportion regression model was used to estimate the hazard ratio (HR) and 95%CI, where survival was set as dependent variable and independent variables included CD4^+^CD25^+^ proportion of T cells, age, progesterone receptor, chemotherapy, endocrine-therapy, and radiotherapy. All comparisons were conducted two-tailed tests with significant level of 0.05.

## CONCLUSIONS

As intra-tumor infiltrating T cells, peripheral blood of CD4^+^CD25^+^ T cells could be regarded as an alternative biomarker to predict the clinical outcomes among whom exposure to DC-CIK infusions, especially among early stage and TNBC patients.
